# Supplementary material for: Widespread allele-specific topological domains in the human genome are not confined to imprinted gene clusters
Source: Genome Biol. 2023 Mar 3;24:40. doi: 10.1186/s13059-023-02876-2 (PMC9983196; doi:10.1186/s13059-023-02876-2)
Supplement: Supplementary file 1 — Additional file 1: Table S1. Capture Regions for RC-HiC in 1_7HB2 Cells. Table S2. Allele Specific Gene Expression Data. Table S3. Allele Specific Methylation Data. Table S4. Core 15-state model (5 marks). Table S5. LOLA Enrichment “Max Rank” Score among 20 genomic features. Table S6. Oligo primers used in qPCR. Table S7. Proportion of ASTAD / non-ASTAD domains overlapping Normal CNV. Table S8. Observed vs. Expected Overlap of Features in Conserved ASTADs. Table S9. Observed vs. Expected Overlap of Genes in ASTADs. [file 13059_2023_2876_MOESM1_ESM.pdf]

Table S1: **Capture Regions for RC-HiC in 1_7HB2 Cells**

ID	Chrom	Start	End	Length	Probes	Probe coverage	Coverage
	**Imprinted Loci**						
**CR_1**	1	65834316	70234314	4399998	6283	753960	379
**CR_2**	14	99726864	102563452	2836588	1431	171720	117
**CR_3**	15	24122197	25769629	1647432	2286	274320	312
**CR_4**	11	1928831	3244496	1315665	1979	237480	61
**CR_5**	8	139610266	140434020	823754	1420	170400	284
	**Non-Imprinted Loci**						
**CR_6**	6	159931187	160466562	535375	857	102840	309
**CR_7**	10	66560359	70694482	4134123	6112	733440	479
**CR_8**	4	103146684	107279803	4133119	5291	634920	335
**CR_9**	2	72046277	76107953	4061676	5829	699480	472
**CR_10**	5	140766361	141459373	693012	1187	142440	563
**CR_11**	6	33182626	33576220	393594	351	42120	30
**CR_12**	1	16567503	16838375	270872	473	56760	48
**CR_13**	2	175906814	176155272	248458	443	53160	337
**CR_14**	11	5200699	5301927	101228	105	12600	235
**CR_15**	7	27105890	27200621	94731	251	30120	949
**CR_16**	6	26183471	26200857	17386	41	4920	533

Details of selected capture regions and oligonucleotide probe coverage for each region. During the analysis each region was analysed independently, such that only HiC read pairs mapped within the same capture region were considered. Ordered by capture region size. Coordinates refer to genome build GRCh38/hg18.

Table S2: **Allele Specific Gene Expression Data**

Dataset	Total	Retained	Source	SNP Source
**GM12878**	482	480	PMID: 31740818	NA
**IMR-90**	455	409	GSM438363	GSM432687
**H1-hESC**	2703	2398	GSM438361	GSM432685

Note: Allele specific gene expression (ASEG) data for GM12878 was obtained from previously published work as a list of 3592 Ensembl gene IDs which were found to contain at least two haplotype informative variants (1). Genes were filtered to retain only those genes (482) with allele specific expression bias (either paternal or maternal). The filtered gene list was merged with Ensembl IDs from Gencode v38. ASEG data for IMR90 and H1-hESC were obtained from the UCSD Human Reference Epigenome Mapping Project (2) via the ASMdb (3). Genes were downloaded as a list of Gene symbols, which were mapped back to their associated Ensembl ID in Gencode v38. A number of genes had ambiguous or unknown Ensembl ID mappings and these were excluded from the final datasets.

Table S3: **Allele Specific Methylation Data**

Dataset	ASM Sites	ASM in CpG	Source	Source Size (Gbp)
**GM12878**	112910	865	GSM1002650	120.4
**IMR-90**	13496	2355	GSM2210597	70.7
**H1-hESC**	126023	656	GSM1002649	127.9

Table S4**: Core 15-state model (5 marks)**

State	Mnemonic	Description
**1**	TssA	Active TSS
**2**	TssAFlnk	Flanking Active TSS
**3**	TxFlnk	Transcription at gene 5' and 3'
**4**	Tx	Strong transcription
**5**	TxWk	Weak transcription
**6**	EnhG	Genic enhancers
**7**	Enh	Enhancers
**8**	ZNF/Rpts	ZNF genes & repeats
**9**	Het	Heterochromatin
**10**	TssBiv	Bivalent / Poised TSS
**11**	BivFlnk	Flanking Bivalent TSS / Enhancer
**12**	EnhBiv	Bivalent Enhancer
**13**	ReprPC	Repressed PolyComb
**14**	ReprPCWk	Weak Repressed PolyComb
**15**	Quies	Quiescent / Low

Note: Data was obtained, for each cell line, from the Roadmap Epigenomics Project (2). 15 states were imputed based on 5 epigenetic marks using chromHMM (4).

Table S5: **LOLA Enrichment “Max Rank” Score among 20 genomic features**

Feature	GM12878	H1-hESC	IMR-90
**A Compartment**	**4**	**9**	**3**
**Weak Transcription**	**7**	13	**3**
**Weak Repressed Polycomb**	11	**10**	14
**CpG Islands**	12	9	15
**lncRNA**	15	**6**	11
**Flanking Active TSS**	**5**	16	**8**
**Repressed Polycomb**	17	**12**	16
**Heterochromatin**	**8**	**5**	17
**Bivalent Enhancer**	18	**13**	**15**
**Enhancers**	**3**	18	**3**
**ZNF / Genes Repeats**	**14**	18	**16**
**Protein Coding**	13	19	17
**Normal (CNV)**	18	**8**	19
**Flanking Bivalent TSS**	19	**15**	19
**Strong Transcription**	**12**	20	**11**
**Genic Enhancer**	15	20	**14**
**Bivalent Poised TSS**	21	16	22
**Quiescent**	22	14	21
**Transcription at 5’ - 3’**	17	22	**20**
**Active TSS**	**9**	22	13
**Deletion (CNV)**	22	21	23
**B Compartment**	23	23	23
**Gain (CNV)**	23	23	**18**
**Loss (CNV)**	24	24	24

Note: Enrichment of genomic features in ASTADs relative to TADs using LOLA (5). Feature labelled according to their max LOLA ranking. Significant enrichment highlighted bold (alpha = 0.01, FDR corrected).

Table S6: **Oligo primers used in qPCR**

Target Gene	5'-Forward Primer-3'	5'-Reverse Primer-3'
ATP10A	AGGTTTTGATCCCAATTTCC	CTTATTCTCTGTCAAAGTGCC
CDKN1C	TCTGATCTCCGATTTCTTCG	CTCTTTGGGCTCTAAATTGG
DIO3	CCAGCACATCCTCGACTAC	ACGTCGCGCTGGTACTTAG
DLK1	GCTCTGTGATAGAGATGTTCG	CAGTCCTTTCCCGAGTACC
H19	CAGAACCCACAACATGAAAG	GTAGTGCAGTGGTTGTAAAG
IGF2	GGGTCGTGCCAATTACATTTCAT	CTTGGACTTTGAGTCAAATTGG
KCNQ1	AAGAAGAAATTCCAGCAAGC	GATCCTTGCTCTTTTCTGAG
KCNQ1-OT1	CTTTGCAGCAACCTCCTTGT	TGGGGTGAGGGATCTGAA
MEG3	ATAATGAGTTCCTGACCTGG	GAAACCAACATCCCACATAC
MEG8	CTGCCTCGAATTCTTTCTTG	CTCTAATCTTCTAGAGCCCC
NPAP1	ATTTACACAAAGCACCTGAG	AAAGGGAGACTAGAGGTTTC
PHLDA2	TAAATCACTTGGCCAGTTTG	TTTTGCAGATGACACGATTC
PRH1	CAAGATGCTTCTGATTCTGC	ATCTGATATTACGAGGGGAAC
PRH1-PRR4	ACTTAACACATCATGAAGGC	ATGATCCTACCACTGAACTC
RTL1	AACAGAGAGTAGCAGAAGAG	CAGGTGATTGATGACATAGC
SETD3	CTGTCCGTTACTTTTGATGG	TGCCTTGATATCTCTTGTTG
SLC22A18	AGTGTGTTCGACCTGAAG	ACATGACCATGAAGAGCC
SNRPN	GAGTTCGAAAGATCAAGCC	AGTCATGGATACCAAGTTCTC
SNURF	AAGAGTGTCAGTTGTACCC	CATTGTCTGGTTTTTGCTTG
TAS2R19	GTGTTGTTCTGGTGATACTG	ATTAGGCTCAGAGTAAAGGG
TAS2R20	AGGCTAAGAGTGTAGTTCTG	ACGTTTCCTTCACATTCTTC
TAS2R43	TCATTCTGGTGATGTTGTTG	GTGAAGGGTACTAAGTTTGC
TAS2R46	TTCTGGTGATACTATTGGGG	TCAGAGTGAAGGGAACTAAG
TAS2R50	AAACTCTGATCTCCTTCCTC	AGGTGTGTTTTAGCTTCTTG
UBE3A	GACTCTCACCCAGTTCTATATC	GATCATCATGTCATCTTCCAC
VRK1	AGGTGTACTTGGTAGATTATGG	ATCTTTTGGGGTCTTCTTTG

Table S7: **Proportion of ASTAD / non-ASTAD domains overlapping Normal CNV**

	ASTAD (%)	All TADs (%)
**GM12878**	59	58
**IMR-90**	49	44
**H1-hESC**	76	72

Note: A domain was only considered overlapping if the entire span overlapped a region with Normal CNV. CNV detection was performed using the aligned HiC data by QDNAseq.

Table S8: **Observed vs. Expected Overlap of Features in Conserved ASTADs**

Test Name	Z score	P-value
**Conserved Heterozygotic Variants**	2.13	0.016
**GM12878, Allele Specific Methylation**	1.67	0.048
**H1-hESC, Allele Specific Methylation**	-1.20	0.885
**IMR-90, Allele Specific Methylation**	1.27	0.102

Note: A domain was only considered overlapping if the entire span overlapped a region with Normal CNV. CNV detection was performed using the aligned HiC data by QDNAseq.

Table S9: **Observed vs. Expected Overlap of Genes in ASTADs**

Test Name	Z score	P-value
**GM12878, Imprinted Genes**	4.15	< 0.001
**GM12878, ASE Genes**	4.59	< 0.001
**H1-hESC, Imprinted Genes**	3.08	0.001
**H1-hESC, ASE Genes**	-1.76	0.961
**IMR-90, Imprinted Genes**	5.00	< 0.001
**IMR-90, ASE Genes**	3.50	< 0.001

Note: The observed number of genes of interest (i.e. Imprinted) overlapping ASTADs was compared against an expected value determined by random sampling of all relevant genes (see methods). A null distribution of repeat samplings (n = 10,000) was built and a Z-score calculated to determine enrichment in ASTADs vs. TADs.

References:

1. Workman RE, Tang AD, Tang PS, Jain M, Tyson JR, Razaghi R, et al. Nanopore native RNA sequencing of a human poly(A) transcriptome. Nat Methods. 2019;
2. Kundaje A, Meuleman W, Ernst J, Bilenky M, Yen A, Heravi-Moussavi A, et al. Roadmap Epigenomics Consortium: Integrative analysis of 111 reference human epigenomes. Nature. 2015;
3. Zhou Q, Guan P, Zhu Z, Cheng S, Zhou C, Wang H, et al. ASMdb: a comprehensive database for allele-specific DNA methylation in diverse organisms. Nucleic Acids Res. 2022;50(D1).
4. Ernst J, Kellis M. ChromHMM: Automating chromatin-state discovery and characterization. Vol. 9, Nature Methods. 2012.
5. Sheffield NC, Bock C. LOLA: Enrichment analysis for genomic region sets and regulatory elements in R and Bioconductor. Bioinformatics. 2016;32(4):587–9.
