# Supplementary material for: Widespread allele-specific topological domains in the human genome are not confined to imprinted gene clusters
Source: Genome Biol. 2023 Mar 3;24:40. doi: 10.1186/s13059-023-02876-2 (PMC9983196; doi:10.1186/s13059-023-02876-2)
Supplement: Supplementary file 2 — Additional file 2: Fig. S1. HiCFlow pipeline and Region Capture HiC (RC-HiC) library. Fig. S2. Comparison of subtraction matrices, at IGF2-KCNQ1 locus, between experimental validated and HiCFlow inferred haplotype in GM12878. Fig. S3. Supporting information relevant to Fig. 2: DNA methylation data and expression levels of imprinted genes. Fig. S4. The effect of the PWS-AS imprinting control region on allele-specific chromatin conformation. Fig. S5. The effect of the IG-DMR/MEG3 imprinting control region on allele-specific chromatin conformation. Fig. S6. Compartment analysis of H19-KCNQ1 and SNRPN loci. Fig. S7. Compartment analysis of DLK1-DIO3 locus. Fig. S8. Features and distribution of ASTADs. Fig. S9. KRT gene cluster on chr12 is within a conserved ASTAD. [file 13059_2023_2876_MOESM2_ESM.pdf]

Fig S1

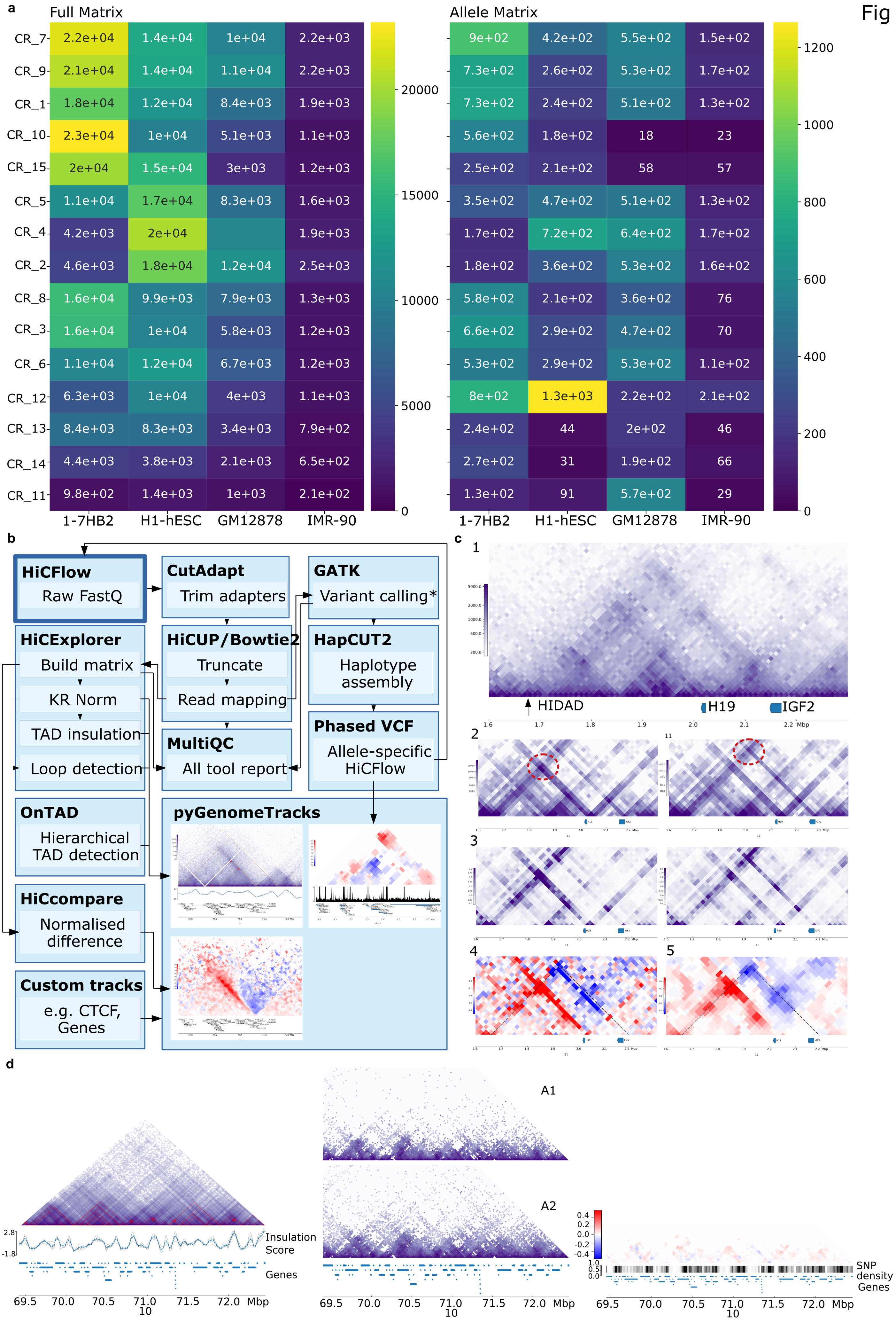
a
Full Matrix
CR_7 2.2e+04 1.4e+04 1e+04 2.2e+03
CR_9 2.1e+04 1.4e+04 1.1e+04 2.2e+03
CR_1 1.8e+04 1.2e+04 8.4e+03 1.9e+03
CR_10 2.3e+04 1e+04 5.1e+03 1.1e+03
CR_15 2e+04 1.5e+04 3e+03 1.2e+03
CR_5 1.1e+04 1.7e+04 8.3e+03 1.6e+03
CR_4 4.2e+03 2e+04 1.9e+03
CR_2 4.6e+03 1.8e+04 1.2e+04 2.5e+03
CR_8 1.6e+04 9.9e+03 7.9e+03 1.3e+03
CR_3 1.6e+04 1e+04 5.8e+03 1.2e+03
CR_6 1.1e+04 1.2e+04 6.7e+03 1.2e+03
CR_12 6.3e+03 1e+04 4e+03 1.1e+03
CR_13 8.4e+03 8.3e+03 3.4e+03 7.9e+02
CR_14 4.4e+03 3.8e+03 2.1e+03 6.5e+02
CR_11 9.8e+02 1.4e+03 1e+03 2.1e+02
1-7HB2 H1-hESC GM12878 IMR-90
20000 15000 10000 5000 0
Allele Matrix
9e+02 4.2e+02 5.5e+02 1.5e+02
7.3e+02 2.6e+02 5.3e+02 1.7e+02
7.3e+02 2.4e+02 5.1e+02 1.3e+02
5.6e+02 1.8e+02 18 23
2.5e+02 2.1e+02 58 57
3.5e+02 4.7e+02 5.1e+02 1.3e+02
1.7e+02 7.2e+02 6.4e+02 1.7e+02
1.8e+02 3.6e+02 5.3e+02 1.6e+02
5.8e+02 2.1e+02 3.6e+02 76
6.6e+02 2.9e+02 4.7e+02 70
5.3e+02 2.9e+02 5.3e+02 1.1e+02
8e+02 1.3e+03 2.2e+02 2.1e+02
2.4e+02 44 2e+02 46
2.7e+02 31 1.9e+02 66
1.3e+02 91 5.7e+02 29
1-7HB2 H1-hESC GM12878 IMR-90
1200 1000 800 600 400 200 0
b
HiCFlow
Raw FastQ
CutAdapt
Trim adapters
GATK
Variant calling*
HiCExplorer
Build matrix
KR Norm
TAD insulation
Loop detection
HiCUP/Bowtie2
Truncate
Read mapping
HapCUT2
Haplotype assembly
MultiQC
All tool report
Phased VCF
Allele-specific HiCFlow
OnTAD
Hierarchical TAD detection
pyGenomeTracks
HiCcompare
Normalised difference
Custom tracks
e.g. CTCF, Genes
c
1
HIDAD
H19
IGF2
1.6 1.7 1.8 1.9 2.0 2.1 2.2 Mbp
2
3
4
5
d
A1
A2
Insulation Score
Genes
SNP density
Genes
69.5 70.0 70.5 71.0 71.5 72.0 Mbp
10

Fig S2

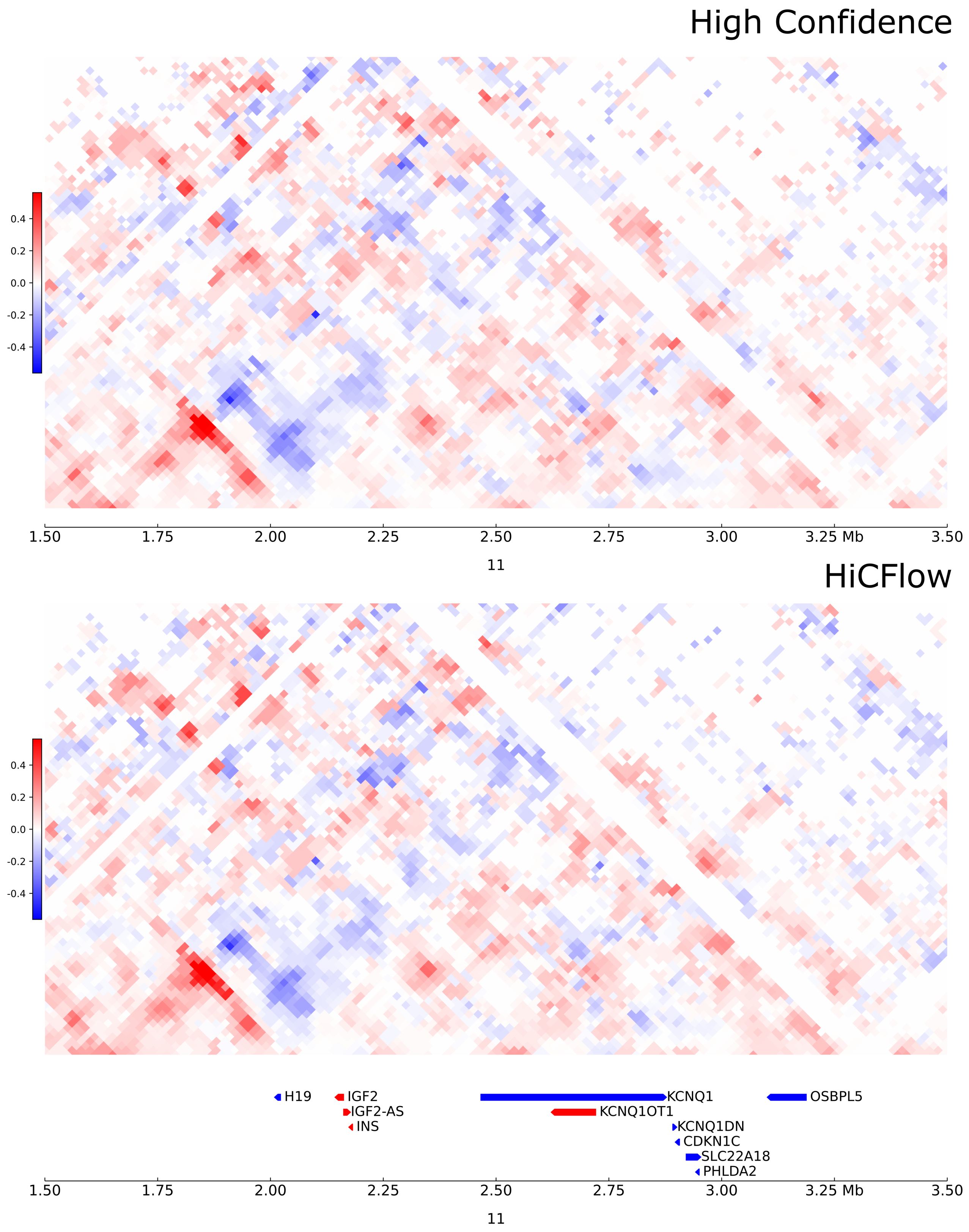
High Confidence
0.4
0.2
0.0
-0.2
-0.4
1.50
1.75
2.00
2.25
2.50
2.75
3.00
3.25 Mb
3.50
11
HiCFlow
0.4
0.2
0.0
-0.2
-0.4
H19
IGF2
IGF2-AS
INS
KCNQ1
KCNQ1OT1
KCNQ1DN
CDKN1C
SLC22A18
PHLDA2
OSBPL5
1.50
1.75
2.00
2.25
2.50
2.75
3.00
3.25 Mb
3.50
11

Fig S3

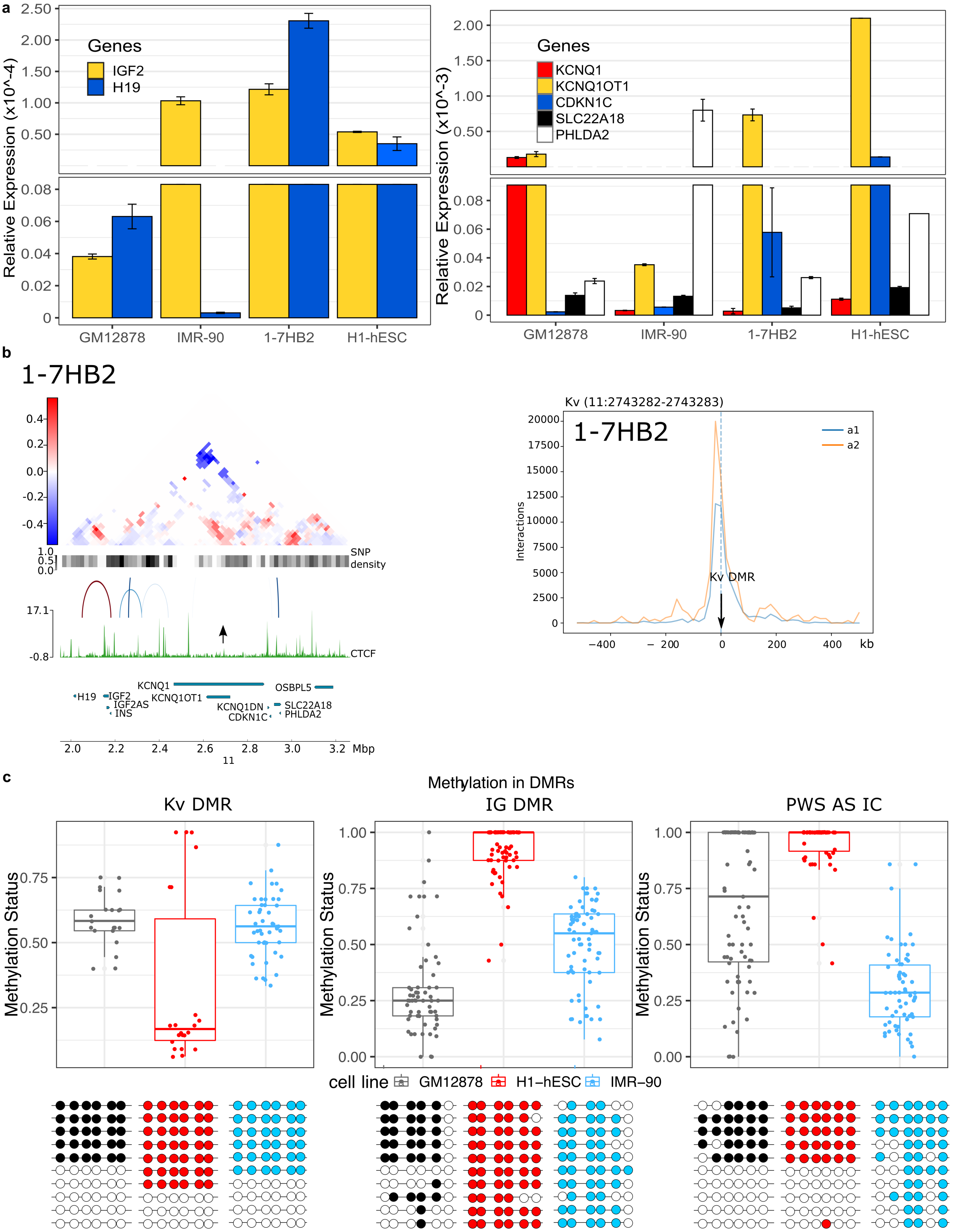
a
Genes
IGF2
H19
Relative Expression (x10^-4)
GM12878
IMR-90
1-7HB2
H1-hESC
Genes
KCNQ1
KCNQ1OT1
CDKN1C
SLC22A18
PHLDA2
Relative Expression (x10^-3)
b
1-7HB2
SNP density
CTCF
KCNQ1
OSBPL5
H19
IGF2
IGF2AS
INS
KCNQ1OT1
KCNQ1DN
CDKN1C
SLC22A18
PHLDA2
Mbp
Kv (11:2743282-2743283)
1-7HB2
a1
a2
Interactions
Kv DMR
kb
c
Methylation in DMRs
Kv DMR
IG DMR
PWS AS IC
Methylation Status
cell line
GM12878
H1−hESC
IMR−90

Fig S4

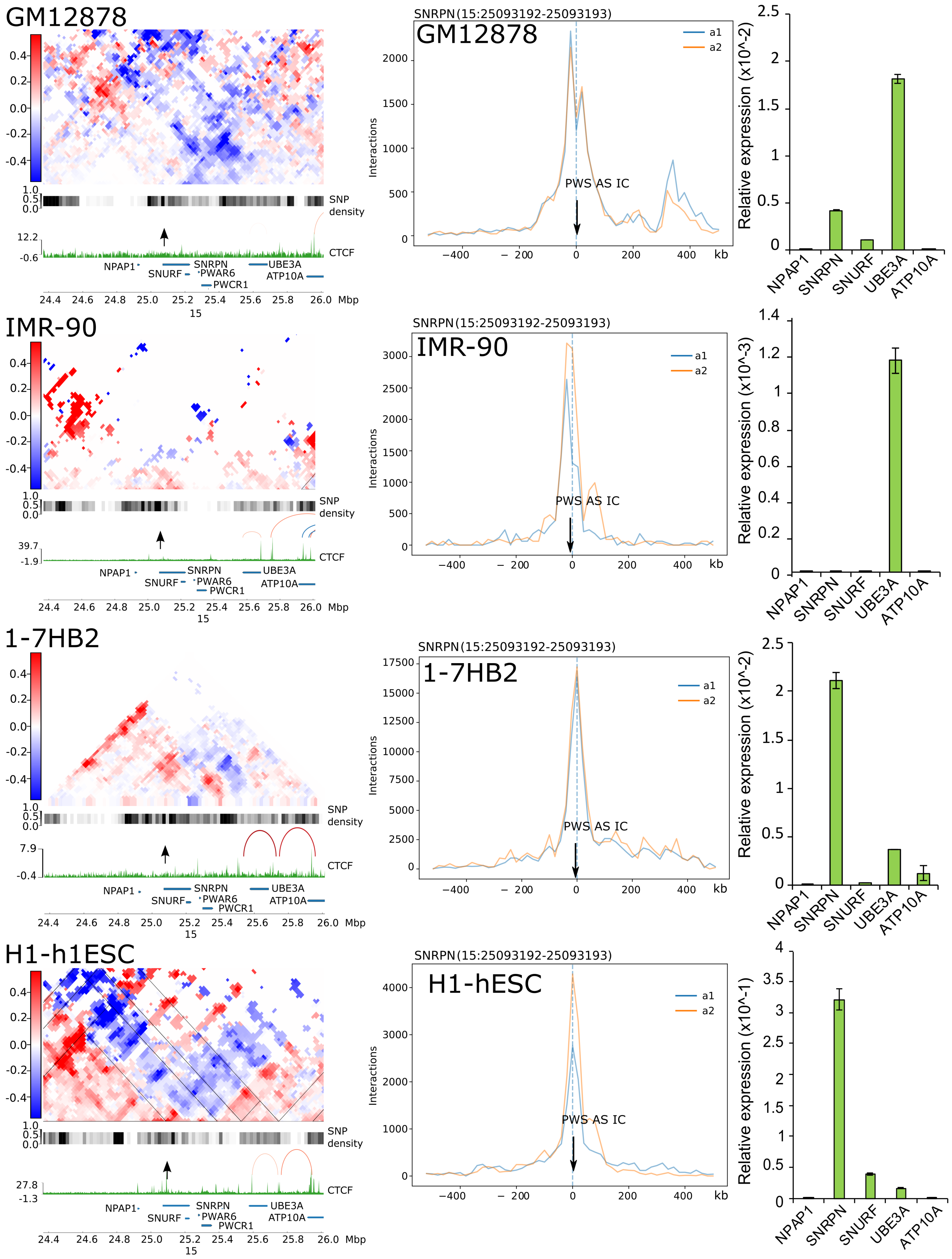
GM12878
IMR-90
1-7HB2
H1-h1ESC
SNP density
CTCF
NPAP1
SNURF
SNRPN
PWAR6
PWCR1
UBE3A
ATP10A
24.4 24.6 24.8 25.0 25.2 25.4 25.6 25.8 26.0 Mbp
15
SNRPN(15:25093192-25093193)
H1-hESC
a1
a2
Interactions
PWS AS IC
kb
Relative expression (x10^-2)
Relative expression (x10^-3)
Relative expression (x10^-1)

Fig S5

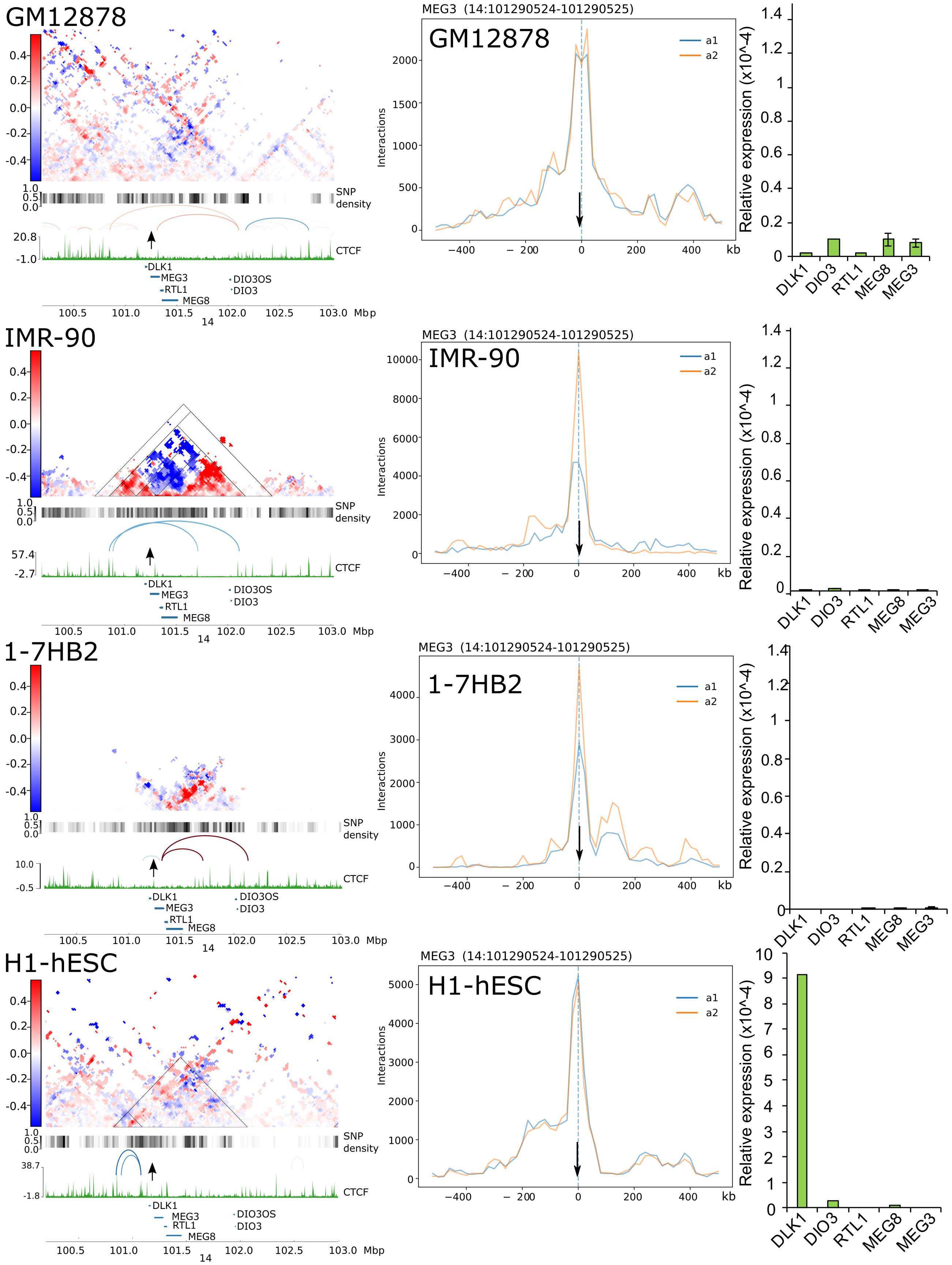
GM12878
0.4
0.2
0.0
-0.2
-0.4
1.0
0.5
0.0
SNP density
20.8
-1.0
CTCF
DLK1
MEG3
RTL1
MEG8
DIO3OS
DIO3
100.5
101.0
101.5
102.0
102.5
103.0 Mbp
14
MEG3 (14:101290524-101290525)
GM12878
a1
a2
Interactions
2000
1500
1000
500
0
−400
− 200
0
200
400
kb
Relative expression (x10^-4)
1.4
1.2
1
0.8
0.6
0.4
0.2
0
DLK1
DIO3
RTL1
MEG8
MEG3
IMR-90
0.4
0.2
0.0
-0.2
-0.4
1.0
0.5
0.0
SNP density
57.4
-2.7
CTCF
DLK1
MEG3
RTL1
MEG8
DIO3OS
DIO3
100.5
101.0
101.5
102.0
102.5
103.0 Mbp
14
MEG3 (14:101290524-101290525)
IMR-90
a1
a2
Interactions
10000
8000
6000
4000
2000
0
−400
− 200
0
200
400
kb
Relative expression (x10^-4)
1.4
1.2
1
0.8
0.6
0.4
0.2
0
DLK1
DIO3
RTL1
MEG8
MEG3
1-7HB2
0.4
0.2
0.0
-0.2
-0.4
1.0
0.5
0.0
SNP density
10.0
-0.5
CTCF
DLK1
MEG3
RTL1
MEG8
DIO3OS
DIO3
100.5
101.0
101.5
102.0
102.5
103.0 Mbp
14
MEG3 (14:101290524-101290525)
1-7HB2
a1
a2
Interactions
4000
3000
2000
1000
0
−400
− 200
0
200
400
kb
Relative expression (x10^-4)
1.4
1.2
1
0.8
0.6
0.4
0.2
0
DLK1
DIO3
RTL1
MEG8
MEG3
H1-hESC
0.4
0.2
0.0
-0.2
-0.4
1.0
0.5
0.0
SNP density
38.7
-1.8
CTCF
DLK1
MEG3
RTL1
MEG8
DIO3OS
DIO3
100.5
101.0
101.5
102.0
102.5
103.0 Mbp
14
MEG3 (14:101290524-101290525)
H1-hESC
a1
a2
Interactions
5000
4000
3000
2000
1000
0
−400
− 200
0
200
400
kb
Relative expression (x10^-4)
10
9
8
7
6
5
4
3
2
1
0
DLK1
DIO3
RTL1
MEG8
MEG3

Fig S6

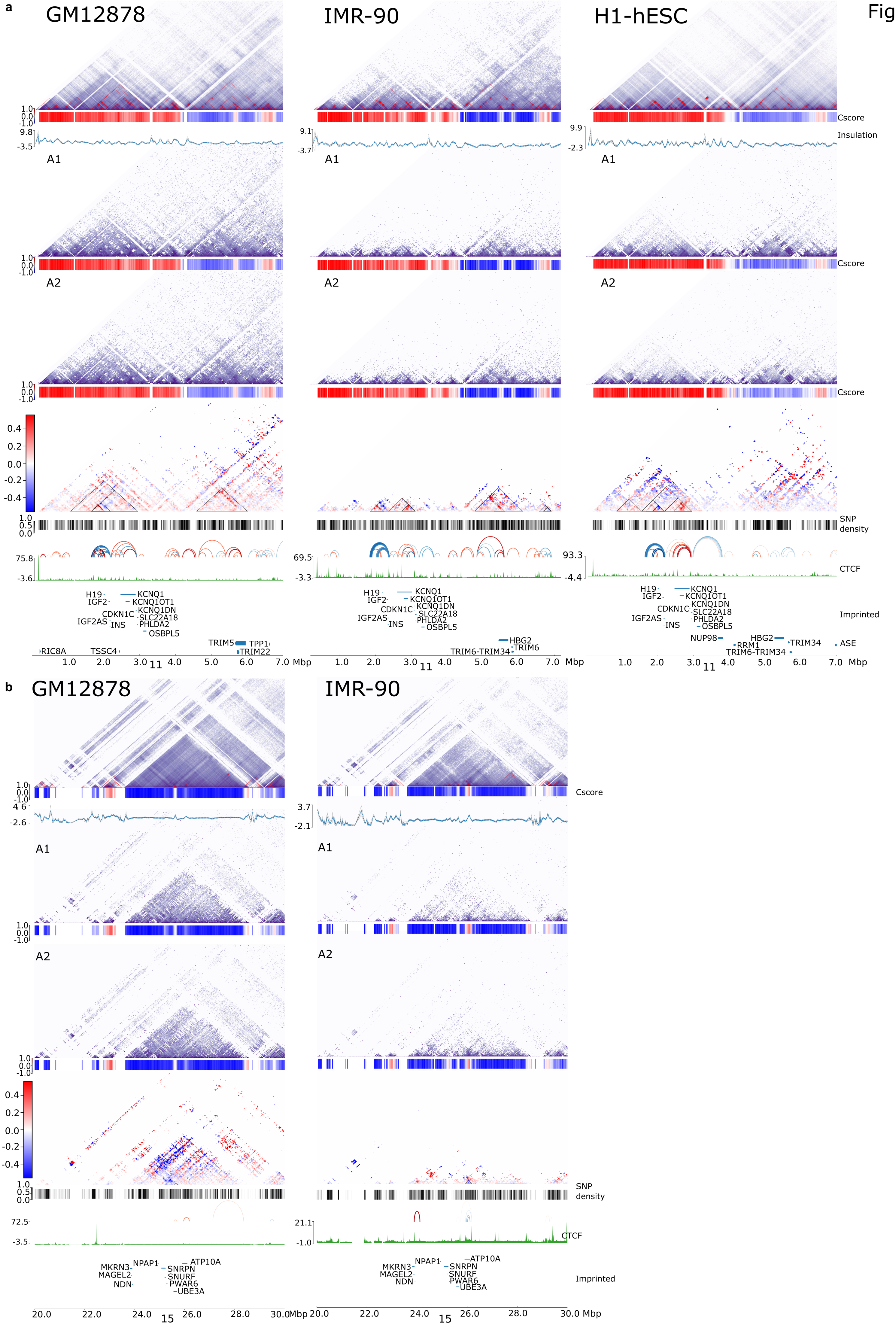

a
GM12878
IMR-90
H1-hESC
Cscore
Insulation
A1
A2
SNP density
CTCF
Imprinted
ASE
H19
IGF2
KCNQ1
KCNQ1OT1
KCNQ1DN
CDKN1C
SLC22A18
IGF2AS
INS
PHLDA2
OSBPL5
TRIM5
TPP1
TRIM22
RIC8A
TSSC4
HBG2
TRIM6
TRIM6-TRIM34
NUP98
RRM1
TRIM34
1.0 2.0 3.0 4.0 5.0 6.0 7.0 Mbp
11
b
GM12878
IMR-90
Cscore
A1
A2
SNP density
CTCF
Imprinted
MKRN3
MAGEL2
NDN
NPAP1
SNRPN
SNURF
PWAR6
UBE3A
ATP10A
20.0 22.0 24.0 26.0 28.0 30.0 Mbp
15

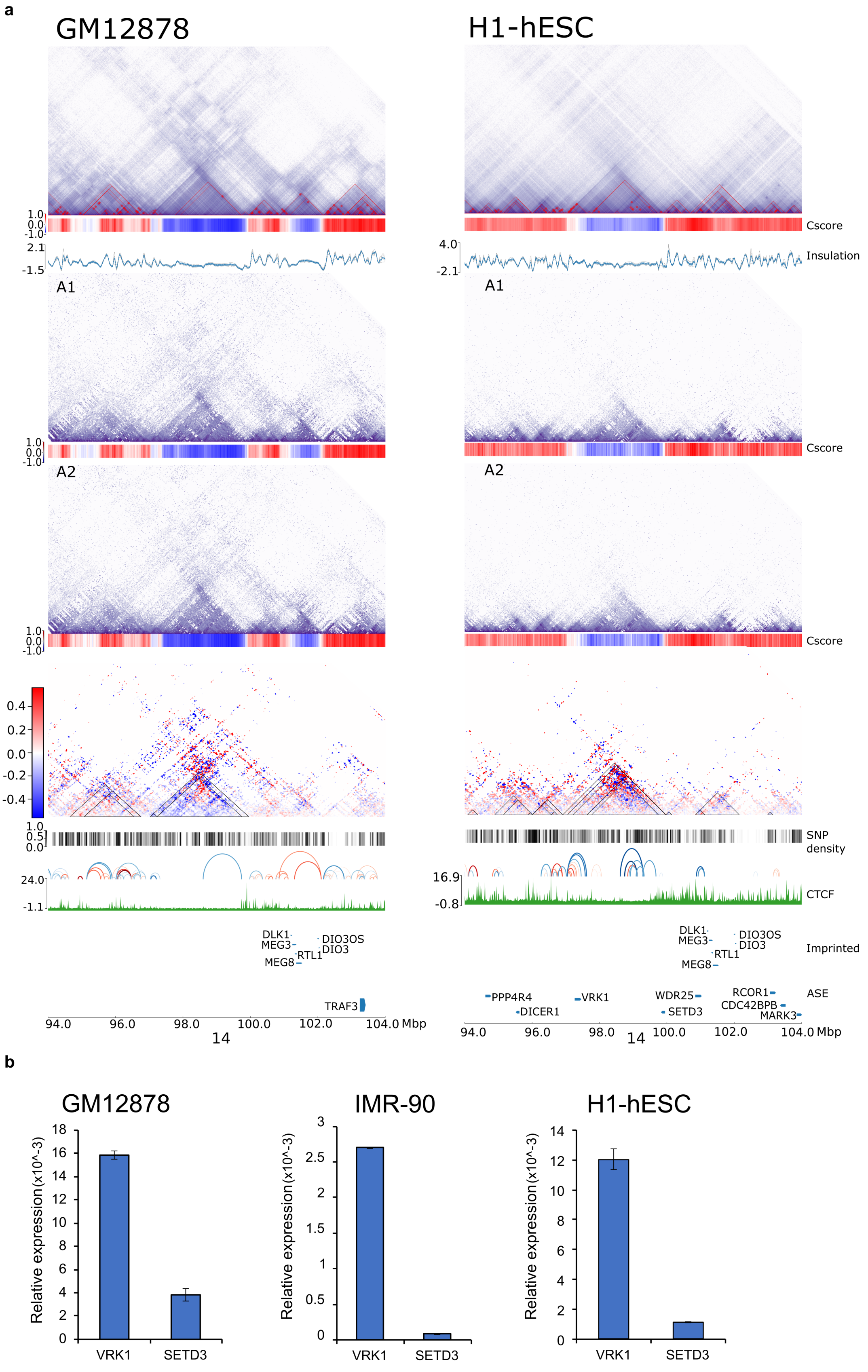
a
GM12878
H1-hESC
Cscore
Insulation
A1
A2
SNP density
CTCF
Imprinted
ASE
DLK1
MEG3
MEG8
RTL1
DIO3OS
DIO3
TRAF3
PPP4R4
DICER1
VRK1
WDR25
SETD3
RCOR1
CDC42BPB
MARK3
94.0
96.0
98.0
100.0
102.0
104.0 Mbp
14
b
GM12878
IMR-90
H1-hESC
Relative expression(x10^-3)
VRK1
SETD3

Fig S8

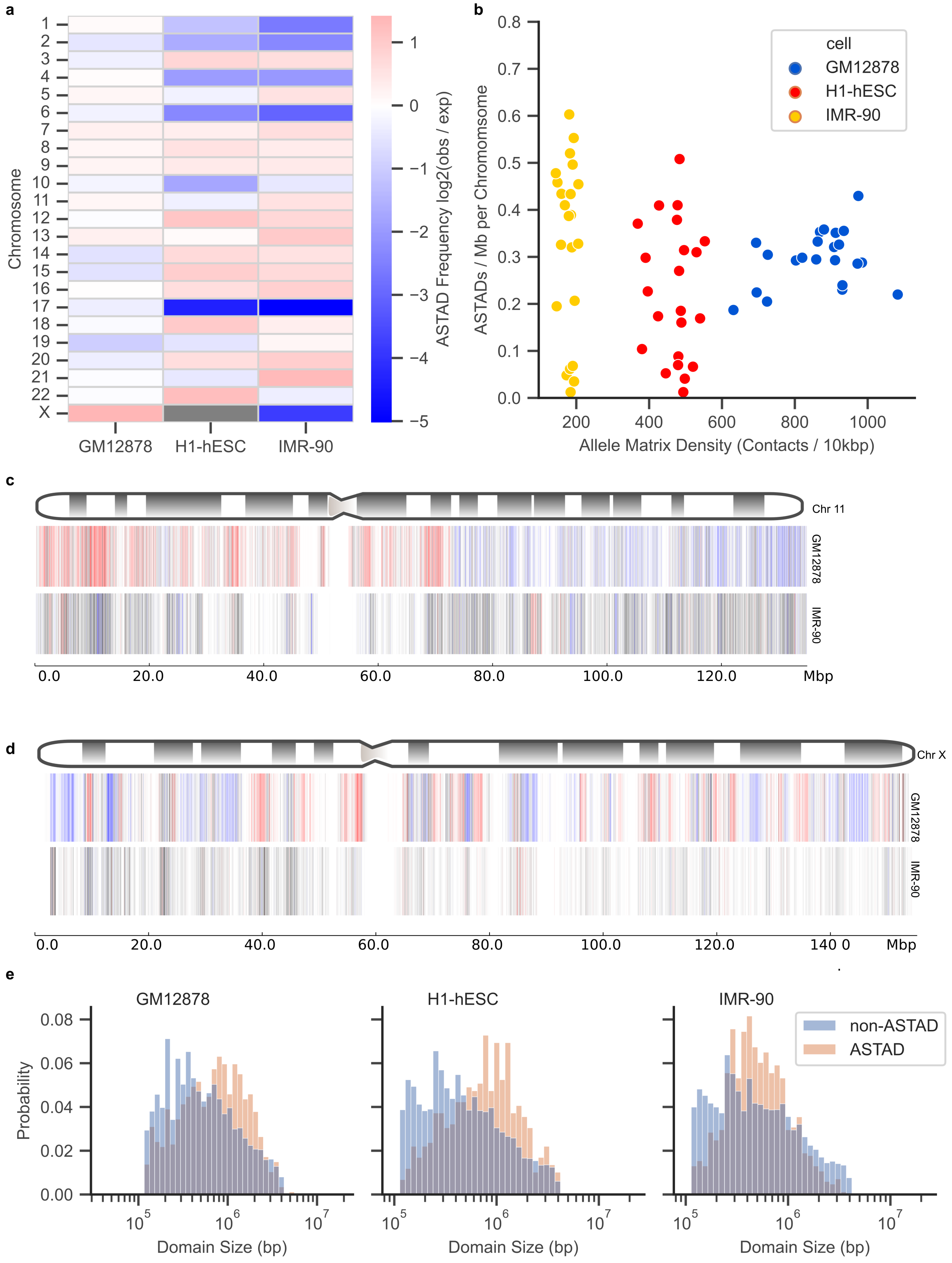
a
Chromosome
1
2
3
4
5
6
7
8
9
10
11
12
13
14
15
16
17
18
19
20
21
22
X
GM12878
H1-hESC
IMR-90
ASTAD Frequency log2(obs / exp)
1
0
−1
−2
−3
−4
−5
b
ASTADs / Mb per Chromomsome
0.8
0.7
0.6
0.5
0.4
0.3
0.2
0.1
0.0
200
400
600
800
1000
Allele Matrix Density (Contacts / 10kbp)
cell
GM12878
H1-hESC
IMR-90
c
Chr 11
GM12878
IMR-90
0.0
20.0
40.0
60.0
80.0
100.0
120.0
Mbp
d
Chr X
GM12878
IMR-90
0.0
20.0
40.0
60.0
80.0
100.0
120.0
140 0
Mbp
e
GM12878
H1-hESC
IMR-90
Probability
0.08
0.06
0.04
0.02
0.00
10^5
10^6
10^7
Domain Size (bp)
non-ASTAD
ASTAD

Fig S9

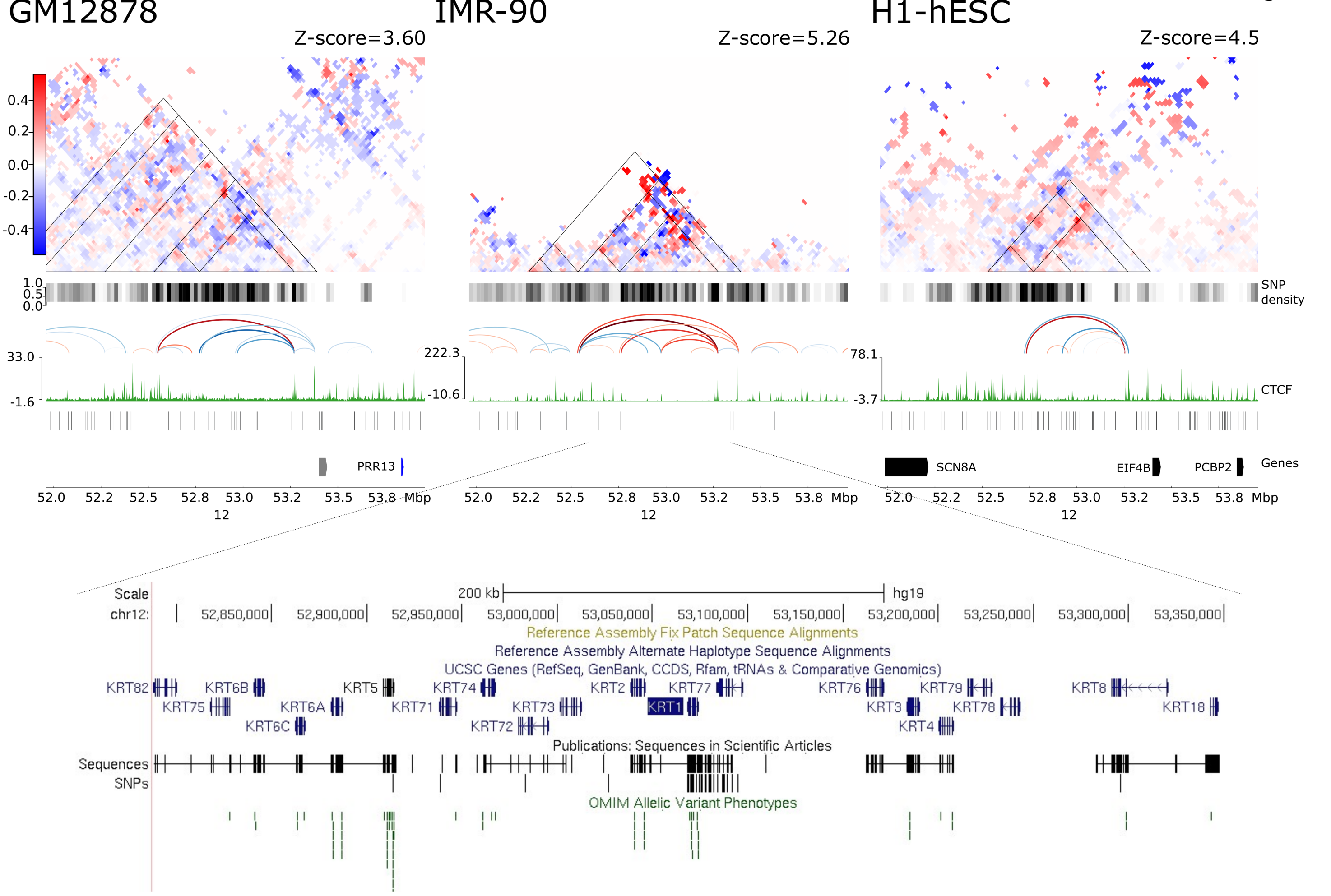
GM12878
Z-score=3.60
IMR-90
Z-score=5.26
H1-hESC
Z-score=4.5
0.4
0.2
0.0
-0.2
-0.4
1.0
0.5
0.0
SNP density
33.0
-1.6
222.3
-10.6
78.1
-3.7
CTCF
PRR13
SCN8A
EIF4B
PCBP2
Genes
52.0 52.2 52.5 52.8 53.0 53.2 53.5 53.8 Mbp
12
Scale
200 kb
hg19
chr12:
52,850,000 52,900,000 52,950,000 53,000,000 53,050,000 53,100,000 53,150,000 53,200,000 53,250,000 53,300,000 53,350,000
Reference Assembly Fix Patch Sequence Alignments
Reference Assembly Alternate Haplotype Sequence Alignments
UCSC Genes (RefSeq, GenBank, CCDS, Rfam, tRNAs & Comparative Genomics)
KRT82
KRT6B
KRT5
KRT74
KRT2
KRT77
KRT76
KRT79
KRT8
KRT75
KRT6A
KRT71
KRT73
KRT1
KRT3
KRT78
KRT18
KRT6C
KRT72
KRT4
Publications: Sequences in Scientific Articles
Sequences
SNPs
OMIM Allelic Variant Phenotypes

Fig S1: HiCFlow pipeline and Region Capture HiC (RC-HiC) library. a) Comparison of mean contact density (contacts/10kb) for RC-HiC (1-7HB2) across capture regions with the highest resolution human HiC datasets available: H1-hESC, GM12878 and IMR-90. In the full diploid matrix, the RC-HiC library yielded more contacts, at the given capture regions. The resolution of RC-HiC allele-specific matrices at the capture regions is comparable to GM12878, for which an experimental validated phased variant truth set is available. The capture region at chr6:26183471-26200857 did not yield sufficient data and was excluded. **b)** Overview of HiCFlow workflow – raw data is processed to publication ready visualisations or may be used for haplotype assembly in conjunction with allele-specific HiC analysis. See Methods for a full description of the bioinformatics tools used for each stage of the workflow. * Variant calling, from the input Hi-C data, is optional and only required if the user has not provided a genotype (.vcf format) to HiCFlow. **c)** Illustration of method for generating subtraction matrix visualisations. The diploid HiC map [1] is split into two haploid matrices [2] using allelic-assignment via mate-rescue (1). Allele specific matrices (A1, A2) are normalised [3] by interaction distance using the method described in HiCExplorer – hicTransform (method=obs_exp) (2). Contact frequencies are subtracted (A2 – A1) [4] and the resulting matrices are de-noised [5], using a median filter (size = 3). This approach facilitates equal comparison of differences at all distances and removal of noisy interactions. **d)** A negative control region at chromosome 10, containing a high density of SNPs, showing that HiCFlow pipeline can split full matrix into haploid alleles (A1 and A2) but does not produce subtraction matrices where there is no allelic differences.

Fig S2: Comparison of subtraction matrices, at *IGF2-KCNQ1* locus, between experimental validated and HiCFlow inferred haplotype in GM12878. No discernable visual differences

were detected between subtraction matrices, indicating appropriateness of using HiCFlow to directly infer haplotype from Hi-C data.

Fig S3: Supporting information relevant to Fig 2: DNA methylation data and expression levels of imprinted genes. a) Quantitative RT-PCR analysis of expression of imprinted genes inside of *H19-IGF2* and *KCNQ1* loci at the four cell lines considered in this study. **b)** 1-7HB2 data for KvDMR effect on allele specific associations showing subtraction matrix between A1 and A2 alleles and viewpoint analysis **c)** Overall methylation states of CpG islands overlapping the imprinting control DMRs (top), determined from WGBS data. Circle plots of representative portions of methylation patterns at the corresponding DMRs obtained by clonal bisulphite analysis. Dark or pale circles represents methylated or unmethylated CGs respectively (bottom). See methods for coordinates of CpGs. The overall methylation plots show the average methylation in the cell line analysed bioinformatically, while the circle plots are an independent experimental validation showing the split of methylation between alleles.

Fig S4: The effect of the PWS-AS imprinting control region on allele-specific chromatin conformation. Denoised subtraction matrix (left) and viewpoint analysis (middle) at the *SNRPN* locus, with underlying SNP density bars, CTCF tracks, allele-specific loops and imprinted gene tracks as in Fig 2. The ICR position is labelled with an arrow above the CTCF track, the coordinates are given above the viewpoint plots. Quantitative RT-PCR analysis of expression of imprinted genes at the *SNRPN* locus (right) in the four cell lines considered in this study.

Fig S5: The effect of the IG-DMR/*MEG3* imprinting control region on allele-specific chromatin conformation. Denoised subtraction matrix (left) and viewpoint analysis (middle) at the *DLK1-DIO3* locus, with underlying SNP density bars, CTCF tracks, allele-specific loops and imprinted gene tracks as in Fig 2. The ICR position is labelled with an arrow above the CTCF track, the coordinates are given above the viewpoint plots. Quantitative RT-PCR analysis of expression of imprinted genes at the *DLK1-DIO3* locus (right) in the four cell lines considered in this study.

Fig S6: Compartment analysis of *H19-KCNQ1* and *SNRPN* loci. Full diploid and phased haploid (A1, A2) Hi-C matrices, with C-sores below indicating combined and allele-specific A-compartments (red) and B-compartments (blue) for wider regions around the imprinted domains. Subtraction matrices showing overall allelic differences, with SNP density, allele specific loops, CTCF tracks, imprinted gene positions as well as an additional track showing genes reported to have allele-specific expression in these cell lines. **a)** *H19-KCNQ1*, **b)** *SNRPN*.

Fig S7: Compartment analysis of *DLK1-DIO3* locus. Full diploid and phased haploid (A1, A2) Hi-C matrices, with C-sores below indicating combined and allele-specific A-compartments (red) and B-compartments (blue) for wider regions around the imprinted domains. Subtraction matrices showing overall allelic differences, with SNP density, allele specific loops, CTCF tracks, imprinted gene positions as well as an additional track showing genes reported to have allele-specific expression in these cell lines. **a)** *DLK1-DIO3* (right) loci in GM12878, IMR-90 and H1-hESC. **b)** Quantitative RT-PCR analysis of expression of *VRK1* and *SETD3* genes in GM12878, IMR-90 and H1-hESC cells.

Fig S8: Features and distribution of ASTADs. a) ASTAD frequency relative to expected TAD proportions per chromosome. In GM12878, autosomal ASTAD distribution is similar to TAD distribution. The X-chromosome has a higher than expected proportion of ASTADs, likely due to skewed X-inactivation. In IMR-90 and H1-hESC ASTAD distribution is more variable with lower than expected proportions of ASTADs observed in chromosomes 1, 2, 4, 6 and 17. Lower than expected ASTADs on chromosome X in IMR-90 is consistent with random X-inactivation, presumably as it was derived from primary lung tissue rather than single cell cloning. **b)** Allelic contact frequency per-chromosome between cell lines. **c)** Ideogram of chromosome 11, with per-bin allelic differences for GM12878 and IMR-90. GM12878 chr11 is involved in a chromosome translocation leading to duplication of part of the q-arm. The ASTAD (A1-A2) change score is high along the entire region in GM12878, while in IMR-90 far fewer regions are highlighted. Regions with copy number changes have been excluded from ASTAD enrichment analysis. **d)** Ideogram of chromosome X with per-bin allelic differences for GM12878 and IMR-90 cells. GM12878 has non-random X-inactivation and shows substantial allelic differences along the chromosome. IMR-90, has random X-inactivation and HiCFlow does not the detect allele-specific differences in a whole cell population. **e)** Histogram of TAD / ASTAD domain sizes. ASTADs domains sizes are comparable to TADs, although there are lower proportions of smaller ASTADs.

Fig S9: *KRT* gene cluster on chr12 is within a conserved ASTAD. Denoised subtraction matrices for the three cell lines showing ASTADs outlined as triangles. The expanded gene panel shows positions of the various *KRT* genes and density of OMIM allelic variant

phenotypes within the conserved ASTAD (see Additional file 7: File S5 for conserved variants at the ASTAD boundaries). Coordinates refer to genome build GRCh37/hg19.

References:

1. Krueger F, Andrews SR. SNPsplit: Allele-specific splitting of alignments between genomes with known SNP genotypes. F1000Res. 2016;5:1479.
2. Ramírez F, Bhardwaj V, Arrigoni L, Lam KC, Grüning BA, Villaveces J, et al. High-resolution TADs reveal DNA sequences underlying genome organization in flies. Nat Commun [Internet]. 2018 Dec 15 [cited 2019 Aug 22];9(1):189. Available from: http://www.nature.com/articles/s41467-017-02525-w
