# Supplementary material for: Widespread allele-specific topological domains in the human genome are not confined to imprinted gene clusters
Source: Genome Biol. 2023 Mar 3;24:40. doi: 10.1186/s13059-023-02876-2 (PMC9983196; doi:10.1186/s13059-023-02876-2)
Supplement: Supplementary file 8 — Additional file 8. Review history. [file 13059_2023_2876_MOESM8_ESM.docx]

Review History

**First round of review**

**Reviewer 1**

**Were you able to assess all statistics in the manuscript, including the appropriateness of statistical tests used?**

No, I do not feel adequately qualified to assess the statistics.

**Comments to author:**

In their paper, Richer et al. investigate 3D genome conformation at human genomic loci that are transcriptionally regulated in an allele-specific manner. For this, they develop a new analysis pipeline that allows haplotype phasing of Hi-C data to generate allele-specific contact maps. Using this workflow, they investigate genome topology at imprinted loci, painting a complex picture between allele-specific genome structure and transcriptional output. They also screen various human cell lines for allele-specific TADs (referred to as ASTADs), revealing that around 10-30% of genes expressed in an allele-specific manner reside in ASTADs. The described bioinformatics pipeline is useful to the field, and the descriptive analyses of allele-specific 3D genome conformation in several frequently-used model cell lines may hold some value for specialist labs in working on genomic imprinting.

Specific comments:

1. The introduction section is unnecessarily long, please consider shrinking it down to the essentials to improve readability of the manuscript. Similarly, the Discussion contains quite some repetition of the Results section, which makes the paper a rather tough read.

2. Overall the study is technically sounds and of sufficient interest. However, its impact could be substantially increased by adding a functional experiment in which an ICR is removed via genome editing, followed by (RC-)Hi-C to assess the true impact of an ICR on allele-specific chromosome conformation. With their current data, all the authors can do is infer that interactions from an ICR are relevant for locus structure and transcription, but this will be just based on correlations.

**Reviewer 2**

**Were you able to assess all statistics in the manuscript, including the appropriateness of statistical tests used?**

Yes, and I have assessed the statistics in my report.

**Comments to author:**

Imprinting is a well described epigenetic phenomenon that results in a parent-of-origin allele specific expression. Alterations in expression of imprinting genes (loss of imprinting), has been associated with human disorders and is frequent in cancer. The role of methylation in establishing and maintaining imprinting is well documented. In addition, CTCF is thought to play a critical role in the regulation of imprinted genes, through allele-specific binding at imprinting control regions, as described in the IGF2/H19 imprinted region. Since CTCF is involved in chromatin organization, a pipeline to detect allele-specific chromatin interactions in imprinted regions to better understand the underlying mechanisms of imprinting maintenance, as well as the functional effects of loss of imprinting in human diseases and cancers is of considerable interest.

In this paper, the authors describe a new bioinformatic pipeline, "HiCFlow", integrating haplotype phasing, allele specific interaction calling and visualization. The pipeline has been tested on capture Hi-C data generated from 1 human breast epithelial cell line, 1-7HB2 and on 3 publicly available Hi-C datasets (GM12878, IMR-90 and H1-ESC). Capture Hi-C was designed to target 3 imprinted loci, IGF2-H19, DLK1 and SNRPN. Hi-C data was used to assess allele-specific interaction genome-wide and beyond imprinted regions. Although this reviewer acknowledges the usefulness of a workflow assessing allele-specific interaction, major concerns were identified throughout the manuscript.

Major comments:

1. For allele-specific approaches, accurate genotyping and haplotype phasing is of importance. While for GM12878, the authors took advantage of public and high confidence phased genotype data, for the other cell lines, genotyping and haplotype phasing were performed using HiCFlow.

A major caveat in the described pipeline is that genotyping was performed directly on raw Hi-C data before phasing using HapCUT2. Hi-C experiments involved a biotin/streptavidin pull-down of cross-linked DNA fragments, which result in a library of ligation products highly enriched in interacting DNA fragments. In the presence of allele-specific interactions (which is the primary event assessed with HiCFlow), DNA fragments corresponding to the non-interacting allele will not be pulled down and therefore won't be sequenced. This is important because genotyping for variants at loci with allele-specific interaction will be inaccurate since heterozygous variants (which are necessary to separate allele and phase haplotype) will be called as homozygous. The phasing in HiCFlow relies on HapCUT2. Although HapCUT2 can phase Hi-C data, its authors specified that the input is a list of heterozygous variants identified by WGS not directly using the Hi-C data (PMID: 27940952).

This is true for most of the allele-specific approaches that have been described, including allele-specific TF binding, ATAC and expression where only binding on the open or expressed allele is sequenced. Published pipelines to study allele-specific marks all require genotyping data from non-enriched genomic DNA (WGS, exome-seq or SNP-array). One of the exceptions is allele-specific methylation using methyl-seq where both alleles (methylated and unmethylated) can be sequenced and therefore used to call heterozygous variants after taking into account the CT conversion.

2. To call heterozygotes on non-enriched genomic DNA data, the authors should perform and describe steps to ensure high quality calls. Those steps should include exclusion of variants in blacklisted regions as well as variants in regions that have other mapping issues, such as variants with multiple mappings. The UCSC browser provides an annotated dbSNP dataset with flags for anomalies suggestive of such issues. This can be useful for filtering variants and regions with problems. Of note, "suspicious" variants are in general more likely to be associated with false positive allele-specific marks due to the mapping associated issues. For example, the authors mentioned that their higher ranked conserved ASTAD overlaps a blacklisted region. Variants called in those regions should be excluded beforehand and not included in any subsequent analyses, unless they are validated by long read sequencing.

3-Capture Hi-C was performed in 1-7HB2 to study the role of chromatin interaction in imprinting regions. While the authors acknowledge the major role of CTCF in imprinting and chromatin organization, matching CTCF ChIP-seq data in 1-7HB2 was not performed and instead public data from MCF10-A was used. Although both cell lines are immortalized breast epithelial cell line, differences in genetic background (which might be associated with sequence-dependent allelic imbalance on top of imprinting) might confound the results.

Minor comments:

1. Allele-specific phenomena include two distinct mechanisms: parent-of-origin (imprinting) and sequence-specific allelic imbalance. Both of them rely on the separation of haplotype based on the presence of heterozygous variants and they can be called using the same workflow. However, a clearer distinction should be made and explained in the manuscript, by defining the two phenomenon and explaining their respective underlying mechanisms and functional effects. The manuscript should be reorganized with this distinction in mind. For example, while the background focuses mostly on imprinting, the discussion starts with sequence-specific allelic imbalance and its implication in GWAS. This might lead to confusion to an "allele-specific naïve" reader.

2.The authors should report, as supplemental information, the number of variants that they were able to phase using HAPcut2. While GM12878 Hi-C data is ~300X deep and ensures numerous accurate phased variants, the other Hi-C sets might have lower coverage and yield sparser informative data. This should be taken into account while comparing ASTAD between cell lines. Likewise, the number of informative valid pairs (assigned to one haplotype) should be reported.

3. In Figure 1,2, and 3, it would be helpful to add the CTCF track, insulation score, gene expression and methylation AFTER allele specific separation below each allele-specific matrix. This could help the reader to understand and visualize the effect of ASTAD on gene expression and the role of CTCF and methylation. However, depending on the regions, informative CTCF peaks, methylation and expression for allele-specific analyses might be too sparse or not available

4. Supplemental Fig.2c, methylation status at DMRs should be analyzed and shown in an allele-specific manner. Partial net methylation level can be suggestive of allele-specific methylation but it does not provide conclusive evidence about this since other mechanisms can lead to partial methylation (such as a mixture of cell subpopulation/states, methylation entropy).

5. In general the manuscript is not well organized - too much of the data is in Supplementary Figures. There should be more figures in the main manuscript.

6. In Figure 4 the authors shift to using H iC data as opposed Capture HiC data for their analysis. I had to go to the materials and methods to find this out. This information should be included in the main text and their approach clearly explained.

7. There should be a clear explanation of olfactory genes.

**Authors Response**

**Point-by-point responses to the reviewers’ comments:**

Reviewer #1:

In their paper, Richer et al. investigate 3D genome conformation at human genomic loci that are transcriptionally regulated in an allele-specific manner. For this, they develop a new analysis pipeline that allows haplotype phasing of Hi-C data to generate allele-specific contact maps. Using this workflow, they investigate genome topology at imprinted loci, painting a complex picture between allele-specific genome structure and transcriptional output. They also screen various human cell lines for allele-specific TADs (referred to as ASTADs), revealing that around 10-30% of genes expressed in an allele-specific manner reside in ASTADs. The described bioinformatics pipeline is useful to the field, and the descriptive analyses of allele-specific 3D genome conformation in several frequently-used model cell lines may hold some value for specialist labs in working on genomic imprinting.

*Reply: We thank the reviewer for taking time to review our manuscript and the overall supportive comments.*

Specific comments:

1. The introduction section is unnecessarily long, please consider shrinking it down to the essentials to improve readability of the manuscript. Similarly, the Discussion contains quite some repetition of the Results section, which makes the paper a rather tough read.

*Reply: We have substantially, shortened the introduction and removed much of the detail surrounding genomic imprinting. We have also edited the discussion to remove repetition and highlight the essential findings.*

2. Overall the study is technically sounds and of sufficient interest. However, its impact could be substantially increased by adding a functional experiment in which an ICR is removed via genome editing, followed by (RC-)Hi-C to assess the true impact of an ICR on allele-specific chromosome conformation. With their current data, all the authors can do is infer that interactions from an ICR are relevant for locus structure and transcription, but this will be just based on correlations.

*Reply: We agree that a functional experiment is always going to enhance any study. The experiment suggested by this reviewer to remove an ICR, would have more value if we were to remove the ICR at all of the imprinted clustered investigated. However, the results of our current study suggest that the ICR has the strongest effect on chromatin conformation at the IGF2-H19 locus, and has a lessor and more indirect effect on chromatin conformation at the other loci, where the ICRs are also less well characterised. A further caveat of such an experiment would be that deleting the ICR’s also removes additional regulatory elements and dissection of these alongside the direct and indirect effect on chromatin conformation would be beyond the scope of this study. For the IGF2-H19 locus, where we see the strongest effect of the ICR contribution to allele-specific chromatin conformation, there is already a wealth of literature demonstrating that deletion of the ICR, or its inactivation through DNA methylation, results in loss of imprinting, with over expression of IGF2 causing Beckwith Wiedeman syndrome. It has previously been shown by us and others that allele-specific deletion of CTCF sites in the ICR, result in allele-specific conformational changes, that cells from Beckwith patients with loss of imprinting have allele-specific profiles consistent with "paternalised" conformation structures, and that perturbation of methylation profiles using 5-Azacytidine results in restructuring of the chromatin conformation of this locus. Therefore, the impact of deleting/modifying the ICR on chromatin conformation is known for this locus. We have included a few sentences in the rewritten discussion (page 30) to highlight the known effects of this ICR on chromatin conformation.*

Reviewer #2:

Imprinting is a well described epigenetic phenomenon that results in a parent-of-origin allele specific expression. Alterations in expression of imprinting genes (loss of imprinting), has been associated with human disorders and is frequent in cancer. The role of methylation in establishing and maintaining imprinting is well documented. In addition, CTCF is thought to play a critical role in the regulation of imprinted genes, through allele-specific binding at imprinting control regions, as described in the IGF2/H19 imprinted region. Since CTCF is involved in chromatin organization, a pipeline to detect allele-specific chromatin interactions in imprinted regions to better understand the underlying mechanisms of imprinting maintenance, as well as the functional effects of loss of imprinting in human diseases and cancers is of considerable interest.

In this paper, the authors describe a new bioinformatic pipeline, "HiCFlow", integrating haplotype phasing, allele specific interaction calling and visualization. The pipeline has been tested on capture Hi-C data generated from 1 human breast epithelial cell line, 1-7HB2 and on 3 publicly available Hi-C datasets (GM12878, IMR-90 and H1-ESC). Capture Hi-C was designed to target 3 imprinted loci, IGF2-H19, DLK1 and SNRPN. Hi-C data was used to assess allele-specific interaction genome-wide and beyond imprinted regions. Although this reviewer acknowledges the usefulness of a workflow assessing allele-specific interaction, major concerns were identified throughout the manuscript.

*Reply: We thank the reviewer for taking time to review our manuscript and the overall supportive comments.*

Major comments:

1. For allele-specific approaches, accurate genotyping and haplotype phasing is of importance. While for GM12878, the authors took advantage of public and high confidence phased genotype data, for the other cell lines, genotyping and haplotype phasing were performed using HiCFlow.

A major caveat in the described pipeline is that genotyping was performed directly on raw Hi-C data before phasing using HapCUT2. Hi-C experiments involved a biotin/streptavidin pull-down of cross-linked DNA fragments, which result in a library of ligation products highly enriched in interacting DNA fragments. In the presence of allele-specific interactions (which is the primary event assessed with HiCFlow), DNA fragments corresponding to the non-interacting allele will not be pulled down and therefore won't be sequenced. This is important because genotyping for variants at loci with allele-specific interaction will be inaccurate since heterozygous variants (which are necessary to separate allele and phase haplotype) will be called as homozygous. The phasing in HiCFlow relies on HapCUT2. Although HapCUT2 can phase Hi-C data, its authors specified that the input is a list of heterozygous variants identified by WGS not directly using the Hi-C data (PMID: 27940952).

This is true for most of the allele-specific approaches that have been described, including allele-specific TF binding, ATAC and expression where only binding on the open or expressed allele is sequenced. Published pipelines to study allele-specific marks all require genotyping data from non-enriched genomic DNA (WGS, exome-seq or SNP-array). One of the exceptions is allele-specific methylation using methyl-seq where both alleles (methylated and unmethylated) can be sequenced and therefore used to call heterozygous variants after taking into account the CT conversion.

*Reply: The reviewer, proposes that heterozygous variants may be misidentified as homozygous variants where allele-specific interactions take place. Specifically, due to a reduction in contact frequency, and associated sequenced read-pairs, at the non-interacting loci.*

*We have cited Edge et al, (PMID: 27940952) as this is HapCUT2 the tool we used for phasing. A key quote from that paper is 'Haplotypes for an individual genome at known heterozygous variants can be directly reconstructed from reference-aligned sequence reads derived from whole-genome sequencing (WGS). ' This relates to the reviewer's criticism although the authors have not said variant calling can't be performed using Hi-C. Regarding HapCUT2 the paper also states: 'Additionally, HapCUT2 and HapCUT were the only tools tested that can reasonably phase paired-end data with long insert lengths that result from proximity ligation (Hi-C) sequencing.' Which is why we choose HapCUT2 over other haplotype assemblers.*

*We have given the reviewer’s concern that many allele-specific experiments suffer from a sequence effect where only one allele is read, some serious thought. Our view is that this bias would increase the sparsity of our phased haplotype, but would not lead to incorrect phasing in HiC data. For a heterozygous region to have a large amount of interactions on only one allele and no interactions on the other allele and thus read as homozygous seems unlikely, since in Hi-C data, allele-specific interactions are defined by pairwise interactions and lack of sequencing due to a loss of an interaction shows up as an imbalance in contact frequency between the paternal and maternal allele. To assess the bias of HiCFlow genotyping / haplotyping, we re-analysed the high confidence phased GM12878 dataset using HiCFlow and compared the genotype and haplotype to the experimental validated variants. Of the loci detected in both datasets there was a high level of agreement between variant calls (99.8%) and phasings (99.9%). In addition, visual comparison at the IGF2 H19 locus where there are known allele-specific interactions, revealed no discernible differences between the truth set and our HiCFlow. We have added this analysis to our results section SI Fig 2.*

2. To call heterozygotes on non-enriched genomic DNA data, the authors should perform and describe steps to ensure high quality calls. Those steps should include exclusion of variants in blacklisted regions as well as variants in regions that have other mapping issues, such as variants with multiple mappings. The UCSC browser provides an annotated dbSNP dataset with flags for anomalies suggestive of such issues. This can be useful for filtering variants and regions with problems. Of note, "suspicious" variants are in general more likely to be associated with false positive allele-specific marks due to the mapping associated issues. For example, the authors mentioned that their higher ranked conserved ASTAD overlaps a blacklisted region. Variants called in those regions should be excluded beforehand and not included in any subsequent analyses, unless they are validated by long read sequencing.

*Reply: We acknowledge the variant-calling methodology was poorly described and have updated the methods accordingly. For variant calling we followed the GATK Best Practices pipeline. The quality of raw variant calls was scored against a set of high-confidence variants obtained from the GATK Resource Bundle using GATK VariantRecalibrator. Finally, low quality variants were filtered using a truth sensitivity filter of 99.5%. We have modified the text in the data procession section of the methods p39.*

3-Capture Hi-C was performed in 1-7HB2 to study the role of chromatin interaction in imprinting regions. While the authors acknowledge the major role of CTCF in imprinting and chromatin organization, matching CTCF ChIP-seq data in 1-7HB2 was not performed and instead public data from MCF10-A was used. Although both cell lines are immortalized breast epithelial cell line, differences in genetic background (which might be associated with sequence-dependent allelic imbalance on top of imprinting) might confound the results.

*Reply: We have now included the correct CTCF track for the 1-7HB2 data (obtained from ERX115548, Illumina) in our figures.*

Minor comments:

1. Allele-specific phenomena include two distinct mechanisms: parent-of-origin (imprinting) and sequence-specific allelic imbalance. Both of them rely on the separation of haplotype based on the presence of heterozygous variants and they can be called using the same workflow. However, a clearer distinction should be made and explained in the manuscript, by defining the two phenomenon and explaining their respective underlying mechanisms and functional effects. The manuscript should be reorganized with this distinction in mind. For example, while the background focuses mostly on imprinting, the discussion starts with sequence-specific allelic imbalance and its implication in GWAS. This might lead to confusion to an "allele-specific naïve" reader.

*Reply: We have based on both reviewer’s comments reorganised our discussion and introduction and more clearly define the different types of allele-specific expression. We have also edited the abstract slightly to highlight these two phenomena.*

2.The authors should report, as supplemental information, the number of variants that they were able to phase using HAPcut2. While GM12878 Hi-C data is ~300X deep and ensures numerous accurate phased variants, the other Hi-C sets might have lower coverage and yield sparser informative data. This should be taken into account while comparing ASTAD between cell lines. Likewise, the number of informative valid pairs (assigned to one haplotype) should be reported.

*Reply: The total number of phased variants identified in each cell line were as follows: IMR-90 (2,231,685), H1-hESC (1,643,225) and GM12878 (2,147,688). This information has now been included in the Methods section, p40.*

*In the ASTAD methodology the differences in each TAD are compared the chromosome-wide background observed absolute differences. This approach standardises for inter-dataset differences in sparsity. Specifically, a TAD is called and ASTAD if the absolute differences observed are two standard deviations (z > 2) above the background. The actual threshold of differences will vary between datasets due to differences in sparsity and other uncontrolled properties of the dataset. Thus, the total number of informative valid pairs identified in each cell line were as follows: IMR-90 (0.13e9), H1-hESC (0.44e9) and GM12878 (0.85e9) This information is now on p 40.*

3. In Figure 1,2, and 3, it would be helpful to add the CTCF track, insulation score, gene expression and methylation AFTER allele specific separation below each allele-specific matrix. This could help the reader to understand and visualize the effect of ASTAD on gene expression and the role of CTCF and methylation. However, depending on the regions, informative CTCF peaks, methylation and expression for allele-specific analyses might be too sparse or not available

*Reply: Unfortunately, the allele-specific data available are very sparse and, we believe, insufficiently informative to draw meaningful interpretation from the visualisations. As such we think the trade-off of additional tracks under these tracks may further clutter the figures without adding much to their interpretability.*

4. Supplemental Fig.2c, methylation status at DMRs should be analyzed and shown in an allele-specific manner. Partial net methylation level can be suggestive of allele-specific methylation but it does not provide conclusive evidence about this since other mechanisms can lead to partial methylation (such as a mixture of cell subpopulation/states, methylation entropy).

*Reply: We now present clonal bisulphite sequencing data for each of the DMRs beneath the graphs in this Figure (now SI Fig 3C). We agree that partial net methylation levels are suggestive of allele-specific methylation levels and that all such data need to be interpreted with caution. For the revised ms, we have experimentally addressed this by bisulphite conversion and cloning and sequencing PCR products to determine the methylation levels at allelic level. These clonal bisulphite data look at single alleles and indicate whether alleles are methylated/unmethylated in cis. Together with the original bioinformatic analysis these data can provide evidence for normal imprinting (i.e when clones are either methylated/unmethylated and overall methylation levels are ~50%).*

5. In general the manuscript is not well organized - too much of the data is in Supplementary Figures. There should be more figures in the main manuscript.

*Reply: We have reorganised the manuscript, but respectfully disagree about the amount of data in the Supplementary figures. These data are essential for a comprehensive study, but the most important figs are in the main manuscript.*

6. In Figure 4 the authors shift to using H iC data as opposed Capture HiC data for their analysis. I had to go to the materials and methods to find this out. This information should be included in the main text and their approach clearly explained.

*Reply: We clearly indicated that we were including HiC analysis from Fig 2 onwards in the original manuscript. In the results sections where we referred to Fig 2, we introduced and justified that we were adding HiC data alongside our Capture HiC data. The subsequent figures focused on the HiC data. We added another sentence on page 22 to indicate that we performed an unbiased ranking of all TADs to assess the allelic associations differences genome-wide in GM12878, IMR-90 and H1-hESC cells (Fig 4a).*

7. There should be a clear explanation of olfactory genes.

*Reply: We have provided enough of an explanation of olfactory genes, given that we don’t actually focus on presenting extensive data on the olfactory genes.*

**Second round of review**

**Reviewer 1**

The authors have sufficiently addressed my comments.

**Reviewer 2**

The authors have addressed most of my concerns except for one important comment. I agree that SNPs associated with allele-specific interaction are rare (~1-2 % in GM12878 cell line using the same HIC public dataset) and that the vast majority of SNP and phase calling using HIC data will be accurate. To support this, the authors show a high agreement between SNP calling from GM12878 WGS and SNP calling from HIC. However, the purpose of this pipeline is to detect allele-specific interactions (ASI). When using HIC data to genotype, SNPs associated with strong allele-specific interaction (and therefore high confidence ASI) are more likely to be excluded from the analysis because their definition overlaps with the definition for homozygous SNPs. For example, in the absence of allele specific interaction for a given heterozygous SNP with two alleles (A and B), in the HIC data, we expect to see 50 % of the reads with allele A and 50 % of the reads with allele B. However, where you have a strong allele-specific interaction, you might observe 90% of the allele A and 10 % of allele B, and depending on the cutoff this could be called as homozygous, with allele A representing more than 80% of the reads. A pipeline dedicated to identifying allele-specific interactions should be able to identify SNPs associated with strong allele-specific interactions (including complete imbalance where all the interactions are observed only on one allele). Although these SNPs might be rare, they are also more likely to have a functional relevance. Identifying regulatory SNPs and their target genes to better understand the underlying mechanisms of disease susceptibility is an important challenge in the post-GWAS era. HICflow could be useful for this purpose by identifying SNPs associated with allele-specific interaction, but I would not be confident using this pipeline if it excludes the possibility of inputting a list of heterozygous SNPs called from WGS, or any other non-biased assays before phasing using HIC data. Although there might be a usefulness for running the pipeline when only HIC data are available, the authors should make it clear for users that they might miss some rare but strong allele-specific interactions. This could be important for some scientific questions and this option should be included into the pipeline. Its not clear why they would leave this important option out.

Minor comments:

SI Figure 3C, p17. The result form the BS cloning for IMR-90 was not consistent with the 50% methylation level observed by WGBS. Although, PCR bias could be observed with BS PCR, it is surprising that all reads overlapping the unmethylated allele will drop especially since the PCR does not seem to be biased for the other cell line. One possible technical reason is that there might be a heterozygous SNP on one of the primer pair, affecting the binding of the primer on the alternative allele. According to DbSNP155, there is a common SNP with 4% allele frequent reported which could be on the primer (rs75182612). The authors should check from their genotyping data if this SNP or other more private SNPs in IMR-90 explain this allele-drop during the BS PCR. In addition, the data in S1 Figure 3C top and bottom panels don’t always agree - there is discrepancy between the bisuphite PCR and the WGBS sequencing. There is no explanation for the other examples which appear strange.

Page 6: ‘It is further assumed that TAD boundaries implicitly prevent transcription read-through and constrain the spread of silencing chromatin.’ There are no citations to support this statement about spread of silent chromatin, and in fact this reviewer only knows of examples where removal of a CTCF site at a boundary results in spreading of active marks, rather than the reverse way round. There are many examples of this reported in the literature including Guo and Alt et al., Nature 2011; Narendra and Reinberg et al., Science 2015.

It is somewhat surprising that there is mosaicism of X chromosome inactivation in the cell line, IMR90. Was this not cloned from a single cell? If this is the case, they need to reference the paper where this information is revealed.

The authors identify only 5 imprinted gene that correspond to two imprinted domains (H19 and another one on chromosome 3) in conserved ASTADs which seems very low and also surprising since the Dlk Dio3 is known to have allele specific interactions. There is no explanation about this.

**Authors Response**

**Point-by-point responses to the reviewers’ comments:**

Reviewer #2:

The authors have addressed most of my concerns except for one important comment. I agree that SNPs associated with allele-specific interaction are rare (~1-2 % in GM12878 cell line using the same HIC public dataset) and that the vast majority of SNP and phase calling using HIC data will be accurate. To support this, the authors show a high agreement between SNP calling from GM12878 WGS and SNP calling from HIC. However, the purpose of this pipeline is to detect allele-specific interactions (ASI). When using HIC data to genotype, SNPs associated with strong allele-specific interaction (and therefore high confidence ASI) are more likely to be excluded from the analysis because their definition overlaps with the definition for homozygous SNPs. For example, in the absence of allele specific interaction for a given heterozygous SNP with two alleles (A and B), in the HIC data, we expect to see 50 % of the reads with allele A and 50 % of the reads with allele B.

However, where you have a strong allele-specific interaction, you might observe 90% of the allele A and 10 % of allele B, and depending on the cutoff this could be called as homozygous, with allele A representing more than 80% of the reads. A pipeline dedicated to identifying allele-specific interactions should be able to identify SNPs associated with strong allele-specific interactions (including complete imbalance where all the interactions are observed only on one allele). Although these SNPs might be rare, they are also more likely to have a functional relevance. Identifying regulatory SNPs and their target genes to better understand the underlying mechanisms of disease susceptibility is an important challenge in the post-GWAS era. HICflow could be useful for this purpose by identifying SNPs associated with allele-specific interaction, but I would not be confident using this pipeline if it excludes the possibility of inputting a list of heterozygous SNPs called from WGS, or any other non-biased assays before phasing using HIC data. Although there might be a usefulness for running the pipeline when only HIC data are available, the authors should make it clear for users that they might miss some rare but strong allele-specific interactions. This could be important for some scientific questions and this option should be included into the pipeline. Its not clear why they would leave this important option out.

*Reply: A feature of HiC is that it enables the linking of SNPs over long distances based on the assumption that most interactions are in cis. We have now added a sentence explaining that, where available, the user should provide a list of SNPs generated from a non-biased assay to control for the possibility of missing rare but very strong allele-specific interactions (page 10).*

*Accordingly, we have now updated the HiCFlow pipeline to enable users to provide their own list of SNPs prior to phasing (SI Fig 1). This additional functionality is also clearly documented on the GitHub page. The functionality remains for users to perform genotyping from HiC data or to provide an externally phased list of SNPs to the allele-specific HiC workflow. Thus, HiCFlow can now be used in the three cases:*

*1) if you don't have a genotype then it can genotype + phase from the HiC data,*

*2) if you already have a validated genotype then it can phase the genotype from the HiC data*

*3) if you already have a validated haplotype then it can be used directly in the AS-HiC workflow.*

*We hope this provides sufficient flexibility to effectively utilise the workflow according to the data that is available.*

Minor comments:

SI Figure 3C, p17. The result form the BS cloning for IMR-90 was not consistent with the 50% methylation level observed by WGBS. Although, PCR bias could be observed with BS PCR, it is surprising that all reads overlapping the unmethylated allele will drop especially since the PCR does not seem to be biased for the other cell line. One possible technical reason is that there might be a heterozygous SNP on one of the primer pair, affecting the binding of the primer on the alternative allele. According to DbSNP155, there is a common SNP with 4% allele frequent reported which could be on the primer (rs75182612). The authors should check from their genotyping data if this SNP or other more private SNPs in IMR-90 explain this allele-drop during the BS PCR. In addition, the data in S1 Figure 3C top and bottom panels don’t always agree - there is discrepancy between the bisuphite PCR and the WGBS sequencing. There is no explanation for the other examples which appear strange.

*Reply: We thank the reviewer for pointing this out and acknowledge that a heterozygous SNP in a primer may bias towards one allele. For a SNP in a primer to significantly skew the amplification towards one allele it needs to be at the 3’end. We have checked all our primers including the abovementioned SNP (rs75182612), which is located on chromosome 15 and affects the primer used as in (Peng et al., 2012), within the SNRPN locus. The latter SNP is thus not relevant to the methylation state of the IG-DMR which is on chr14. Thus, for the discrepancy between the IG-DMR clonal analysis and overall methylation states that we observe, we still assume that the clonal analysis may be due to allele drop-out or bisulphite PCR related artefact.*

*At the SNRPN locus, the Rs75182612 SNP does overlap our primer, but the cell lines are all homozygous for this SNP.*

*In the manuscript (page 18) we have highlighted where the experimentally derived circle plots do not directly correlate with the overall levels detected bioinformatically and provided more information on how to interpret the methylation analysis.*

Page 6: ‘It is further assumed that TAD boundaries implicitly prevent transcription read-through and constrain the spread of silencing chromatin.’ There are no citations to support this statement about spread of silent chromatin, and in fact this reviewer only knows of examples where removal of a CTCF site at a boundary results in spreading of active marks, rather than the reverse way round. There are many examples of this reported in the literature including Guo and Alt et al., Nature 2011; Narendra and Reinberg et al., Science 2015.

*Reply: We apologise for omitting to include a reference here. We have now included the citations of Narenda and Reinberg as suggested as well as added two further references (Ulianov et al 2016 and Khoury et al 2020), who highlight long range epigenetic regulation and the constraining of transcriptionally active domains by CTCF mediated TADs (page 6).*

It is somewhat surprising that there is mosaicism of X chromosome inactivation in the cell line, IMR90. Was this not cloned from a single cell? If this is the case, they need to reference the paper where this information is revealed.

*Reply: We have added a reference for IMR-90 cells in the methods section (IMR-90 - CCL-186 | ATCC). These cells were not from a single cell clone but from primary lung tissue, and have a limited number of population doublings, thus maintain their random X-inactivation. We have added a comment to this effect on page 23-24.*

The authors identify only 5 imprinted gene that correspond to two imprinted domains (H19 and another one on chromosome 3) in conserved ASTADs which seems very low and also surprising since the Dlk Dio3 is known to have allele specific interactions. There is no explanation about this.

*Reply: We do not dispute that DLK and DIO3 have allele-specific interactions, and this is shown in Fig1, SI Fig5, and SI Fig7. We respectfully disagree with the reviewer that no explanation was offered for them not being present in a conserved ASTAD. On page 26 of the original manuscript, (now page 27), we noted that there were differences in the frequencies of allele-specific interactions between the different cell lines for KCNQ1, SNRPN and DLK1. At the end of that section, we point out that “Randomisation testing (see methods) revealed that imprinted genes were significantly enriched (p <= 0.001) within ASTADs compared to non-ASTADs (SI Table 9). However, the observation that so few imprinted genes were in conserved ASTADs suggest that somatic differences in imprinted gene expression, methylation, and other epigenetic effects, influence the density of allele-specific interactions around them such that their TADs do not consistently meet the threshold of a conserved ASTAD”.*

**Third round of review**

**Reviewer 2**

The authors have addressed all my comments- except one minor one: Page 6: 'It is further assumed that TAD boundaries implicitly prevent transcription readthrough and constrain the spread of silencing chromatin.' There are no citations to support this statement about spread of silent chromatin, and in fact this reviewer only knows of examples where removal of a CTCF site at a boundary results in spreading of active marks, rather than the reverse way round. There are many examples of this reported in the literature including Guo and Alt et al., Nature 2011; Narendra and Reinberg et al., Science 2015.

*Reply: We apologise for omitting to include a reference here. We have now included the citations of Narenda and Reinberg as suggested as well as added two further references (Ulianov et al 2016 and Khoury et al 2020), who highlight long range epigenetic regulation and the constraining of transcriptionally active domains by CTCF mediated TADs (page 6).*

The words transcriptionally active were not put in the manuscript - instead the authors left the sentence with 'spread of silencing chromatin' which is not correct.

**Authors Response**

**Point-by-point responses to the reviewers’ comments:**

Reviewer #2:

The authors have addressed all my comments- except one minor one: Page 6: 'It is further assumed that TAD boundaries implicitly prevent transcription readthrough and constrain the spread of silencing chromatin.' There are no citations to support this statement about spread of silent chromatin, and in fact this reviewer only knows of examples where removal of a CTCF site at a boundary results in spreading of active marks, rather than the reverse way round. There are many examples of this reported in the literature including Guo and Alt et al., Nature 2011; Narendra and Reinberg et al., Science 2015.

*Reply: We apologise for omitting to include a reference here. We have now included the citations of Narenda and Reinberg as suggested as well as added two further references (Ulianov et al 2016 and Khoury et al 2020), who highlight long range epigenetic regulation and the constraining of transcriptionally active domains by CTCF mediated TADs (page 6).*

The words transcriptionally active were not put in the manuscript - instead the authors left the sentence with 'spread of silencing chromatin' which is not correct.

*Reply: Referee 2’s objection to us having left the phrase “spread of silencing chromatin” in our manuscript, seems to us, (with due respect to the reviewer) somewhat pedantic. Within the field there is an assumption made by several authors that TAD domains separate active and silent domains. There is some research in the drosophila field (Heurteau et al., 2020) and also in the Polycomb field that indicate that the 3D conformation at PRC bound loci strengthens silencing and modulates long range interactions as part of an integral aspect of PRC regulatory function (Gentile & Kmita, 2020). Added evidence comes from the study by Witcher et al. who shows that at the p16 locus, ablation of CTCF results in changes in long range contacts, and apparent spreading of heterochromatin from upstream regions and that the function of the boundary is to maintain the p16 gene in a transcriptionally active chromatin domain (Witcher & Emerson, 2009).*

*We concede that to date it has been easier to experimentally show that disruption of a boundary leads to activation (easily demonstrated by a PCR assay), whereas demonstration of silencing requires further chromatin characterisation experiments. We have now added the above three references to back up the assumption in the field. However, we do not have an appetite to enter a protracted debate about whether spreading of silencing is right or wrong, especially if this will further delay publication. If the editorial board thinks it is essential to comply with this minor reviewer’s comment, we will delete the “spreading of silent chromatin” subphrase and associated new references.*

*Gentile, C., & Kmita, M. (2020). Polycomb Repressive Complexes in Hox Gene Regulation: Silencing and Beyond. BioEssays, 42(10), 1900249. https://doi.org/10.1002/BIES.201900249.*

*Heurteau, A., Perrois, C., Depierre, D., Fosseprez, O., Humbert, J., Schaak, S., & Cuvier, O. (2020). Insulator-based loops mediate the spreading of H3K27me3 over distant micro-domains repressing euchromatin genes. Genome Biology, 21(1), 1–19. https://doi.org/10.1186/S13059-020-02106-Z/FIGURES/7.*

*Witcher, M., & Emerson, B. M. (2009). Epigenetic Silencing of the p16INK4a Tumor Suppressor Is Associated with Loss of CTCF Binding and a Chromatin Boundary. Molecular Cell, 34(3), 271–284. https://doi.org/10.1016/J.MOLCEL.2009.04.001.*
